# The Cultural Supplement: A New Method for Assessing Culturally Relevant Prolonged Grief Disorder Symptoms

**DOI:** 10.32872/cpe.7655

**Published:** 2023-03-31

**Authors:** Clare Killikelly, Andreas Maercker

**Affiliations:** 1 Department of Psychology, University of Zürich, Zurich, Switzerland; 2 Department of Psychiatry, University of British Columbia, Vancouver, Canada; Philipps-University of Marburg, Marburg, Germany

**Keywords:** prolonged grief disorder, ICD-11, International Prolonged Grief Disorder scale, cultural adaptation

## Abstract

**Background:**

The new diagnosis of prolonged grief disorder (PGD) is both an opportunity and a challenge for researchers, clinicians, and bereaved individuals. The latest definition of PGD includes a refreshing and novel feature: the cultural caveat, i.e., clinicians must determine that the grief presentation is more severe and of longer duration than would be expected by an individual’s culture and context. Currently, there are no guidelines on how to operationalize the cultural caveat in mental health care settings.

**Method:**

To respond to this important demand we have developed, piloted, and tested the cultural supplement module of the International Prolonged Grief Disorder scale (IPGDS). The cultural supplement aims to provide clinicians with a catalogue of culturally relevant symptoms of grief that indicate probable PGD alongside a simple framework for cultural adaptation for use in specific clinical settings.

**Results:**

In this short report we outline the rationale and aim of the cultural supplement and provide a summary of our latest validation studies of the IPGDS with bereaved German-speaking, Chinese and Swiss migrant individuals. We also provide a step-by-step framework for adaptation of the cultural supplement that clinicians and researchers may use when working with different cultural groups.

**Conclusion:**

To date, this is the first PGD questionnaire based on the ICD-11, and the first to include a cultural supplement that can be adapted to different contexts and groups. This cultural supplement will provide clinicians and researchers an easy-to-use assessment tool with the aim to improve the global applicability of the ICD-11 PGD definition.

## Highlights

- We explore the role of culture and the new diagnosis of prolonged grief disorder.
- We provide the framework for a new method of cultural adaptation for a grief assessment measure.
- We summarize new research using this new method in different cultural groups around the world.
- We provide key recommendations for clinical practice.

## Prolonged Grief Disorder

In 2022 the latest revision of the ICD-11 was implemented in clinical and research settings around the world. Prolonged grief disorder (PGD) is a new mental health disorder included in the ICD-11. The inclusion of grief as a mental disorder has been hailed as both an opportunity and a challenge for researchers, clinicians, and patients (Bryant, 2014; Killikelly & Maercker, 2017; Stelzer, Zhou, Merzhvynska, et al., 2020; Stroebe et al., 2008). In the latest iteration of the ICD-11 the WHO outlined a new remit for the structure and content for disorder definitions. A strong emphasis on clinical utility and global applicability was prioritized over further delineation of accessory symptoms and subtypes (Keeley et al., 2016). This led to the inclusion of refreshing new features in the diagnostic definition of PGD. The cultural caveat purports that for a diagnosis to be assigned, the symptoms of PGD must be more intense, more severe and of longer duration than would normally be expected for the individuals’ cultural or religious context. This is an exciting and novel feature for a diagnostic definition. It holds the promise of a more inclusive, globally applicable classification system, that may improve diagnostic accuracy, therapeutic rapport and treatment outcomes (Aggarwal, 2013). However, the ICD-11 falls short of providing clear guidance on how to operationalize the cultural caveat. Questions remain about how to differentiate symptoms of ‘normal’ bereavement in different contexts (e.g., child loss, unnatural violent loss, ambiguous loss) alongside ‘disordered’ symptoms in different cultures around the world.

Historically the fields of culture, psychology, and psychiatry have only recently intersected to develop models and frameworks to explore the contribution of culture to psychopathology. Earlier in the history of psychiatry, it had been largely assumed that the symptoms of disorder expressed in North American and European populations were representative globally. Recent research has confirmed that the symptom content and structure, duration, chronicity and response to treatment can be highly dependent on culture (Kohrt et al., 2014; Nichter, 1981). This relativist view of disorder purports that the boundary between normal and abnormal is a social judgment or a social/cultural norm and that the definition of abnormal will change depending on particular culture norms (Canino & Alegría, 2008). There are several examples of how disordered grief may manifest differently in different cultures. Unique culturally bound symptoms of PGD have been identified worldwide (Killikelly et al., 2018; Rosenblatt, 2008). For example, in traumatically bereaved Kurdish refugees one common expression of severe grief was to imitate the behaviours of the deceased (Hall et al., 2014), 52% of Cambodia refugees reported dreams of the deceased and this was associated with elevated PGD symptoms (Hinton et al., 2013), in Japan bereaved individuals will control their grief at funerals as they do not want to make others uncomfortable (Killikelly et al., 2022).

On the other hand, the universalist approach (or pan cultural approach) suggests that mental disorders have core symptoms of internal disorder however these symptoms may manifest differently in different contexts (Canino et al., 1997). A famous study on experiences of grief around the world examined the expression of emotion after bereavement in 78 cultures (Rosenblatt et al., 1976). They concluded that it is a basic human characteristic to react with emotions towards bereavement and for the majority of societies these emotions included crying, overt anger, and fear. In an early study comparing Dutch and Slovenian spouses who lost their partner due to unnatural causes it was found that there were more similarities than differences between cultures (Cleiren et al., 1996). Although in Slovenian people symptoms of depression were slightly higher the overall pattern was very similar. There is a gap in the research field, as currently there are no up to date studies that directly compare symptoms of PGD across cultures, particularly the latest ICD-11 definition of PGD. Additionally, there are no culturally adapted questionnaires or measures of prolonged grief disorder. It is therefore difficult to ascertain if PGD symptoms follow a relativist or universalist trend, especially without adequate assessment measures.

New research is consistently demonstrating the importance in cultural adaptation of mental health assessment interviews and questionnaires (Hall et al., 2016). However currently, there is discourse and debate in the field over the level of cultural adaptation that is required to successfully evaluate mental disorders in cultures outside of Europe and North America (Harper Shehadeh et al., 2016; Heim & Kohrt, 2019). There are currently two broad approaches to the development or adaptation of culturally sensitive mental health tools. The etic approach refers to a questionnaire or intervention developed outside of the culture whereas the emic approach refers to a questionnaire or intervention developed from within a culture (Triandis & Marin, 1983). Both of these approaches have been used in the cultural clinical psychology field to varying degrees (Heim et al., 2017; Killikelly et al., 2018; Rasmussen et al., 2014) and with conflicting results. Some clinicians and researchers argue that the etic approach is enough to provide a clear and valid understanding of disorder, while others argue that the evaluation must stem from within the culture in order to be valid (Aggarwal et al., 2014; Berry, 1969). Currently there is a dearth of both etic or emic approaches to assessment in the field of prolonged grief diagnosis, assessment and treatment. Here we consider both the emic and etic approach in a new combined PGD assessment methodology.

## IPGDS and Cultural Supplement

The International Prolonged Grief Disorder Scale (IPGDS) is a two-part assessment questionnaire. This questionnaire is unique as it includes both emic and etic methodology within one questionnaire. The first part is the ‘standard scale’; a 14-item scale developed directly from the latest narrative definition of PGD from the ICD-11. This represents the etic approach. It contains two core items (longing or yearning for the deceased, preoccupation with the deceased), accessory items including examples of emotional pain, time and impairment criteria and the cultural caveat. The standard scale can be used to determine a preliminary diagnosis of PGD and is a clinical diagnostic tool. The second part of the IPGDS is the cultural supplement and uses an emic approach. The cultural supplement was developed from focus groups and key informant interviews from health care professionals and bereaved individuals from a range of cultural backgrounds. The aim was to collect a catalogue of possible PGD symptoms that may be culturally relevant above and beyond the standard ICD-11 items (Table 1). The cultural supplement is intended to provide a more in-depth assessment of possible PGD symptoms that may improve treatment decision making with clinical guidance. For example, recently a novel study of bereaved Balinese family members revealed a probable caseness of 0% for PGD, 1% for posttraumatic stress disorder and 2% for depression. These findings are striking as usually rates of PGD are expected to be at least around 1% and more commonly less than 10% of the population. The authors conclude that there are perhaps aspects of the Balinese culture that protect individuals from developing mental health disorders (Djelantik et al., 2021). However, another explanation could be that the scale used to measure PGD was not culturally adapted from within the population and instead only used at etic approach. Therefore, the scale may not have captured PGD symptoms that are most distressing or representative in this population. It was recently suggested that PGD assessment across cultures would benefit from the inclusion of both etic and emic methods within an assessment tool, such as provided by the IPGDS (Kokou-Kpolou, 2021). Below is a summary of our research exploring the development and first applications of this combined methodology using both the etic ‘standard scale’ and the emic cultural supplement of the IPGDS in different cultural groups.

**Table 1:** IPGDS Cultural Supplement Items: Developing a Catalogue Culturally Relevant of Grief Symptoms

Cultural Supplement item	German-speaking sample	Chinese sample	Arabic migrant sample
I experience strong physical problems since the loss (e.g., headache, problems with appetite).	**Figure:** 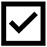	**Figure:** 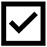	**Figure:** 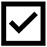
I would do anything to feel close to the deceased (e.g., visit their grave everyday, sleep next to their picture).	**Figure:** 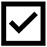		If I could, would do anything to feel close to the deceased (e.g., visit their grave everyday, sleep next to their picture). (slightly reworded item)
Since the loss my behavior has changed drastically in an unhealthy direction (e.g., excessive alcohol consumption).	**Figure:** 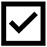		**Figure:** 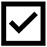
The loss shattered my trust in life or faith in God/a higher spiritual power.	**Figure:** 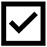		The loss shattered my beliefs (i.e. my understanding of how the world should work, spiritual beliefs, religious beliefs).
It is impossible for me to focus.	**Figure:** 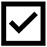	**Figure:** 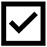	**Figure:** 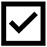
My grief is so intense that I feel stuck in grief.	**Figure:** 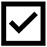	**Figure:** 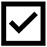	**Figure:** 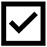
I just can’t seem to fall back into a rhythm.	**Figure:** 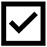		**Figure:** 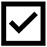
I feel paralyzed and disconnected, (e.g., as if I am not in my own body).	**Figure:** 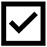	**Figure:** 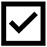	**Figure:** 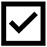
I have no energy or desire to engage in activities.	**Figure:** 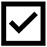		**Figure:** 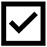
This life holds no meaning since the death.	**Figure:** 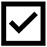	**Figure:** 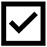	**Figure:** 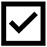
I want to die to be with the deceased.	**Figure:** 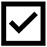	**Figure:** 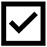	**Figure:** 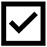
I don’t feel close to other people or feel no satisfaction when being around others.	**Figure:** 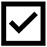		**Figure:** 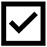
I feel like I have completely lost control.	**Figure:** 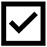		I feel like I have completely lost control over my life or over myself.
I am searching for the deceased with the hope to find him/her.	**Figure:** 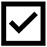	**Figure:** 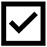	**Figure:** 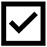
I constantly look back upon the past relationship.		**Figure:** 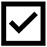	
I feel so helpless since I lost him/her.		**Figure:** 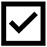	**Figure:** 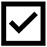
I feel he/she is beside me.		**Figure:** 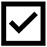	**Figure:** 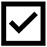
I cry loudly when I think of the loss.		**Figure:** 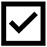	**Figure:** 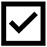
I can’t trust others since the loss.		**Figure:** 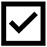	
I feel disconnected from the new society I live in (e.g. the country I move to).			**Figure:** 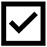
Without a funeral (body, or other burial ritual) I cannot move on with my life.			**Figure:** 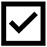 TBC
Not knowing what happened to them is the worst part.			**Figure:** 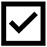 TBC
I would rather know they are dead then face this uncertainty.			**Figure:** 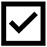 TBC
If I were in my home country I would have more support for my grief.			**Figure:** 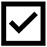 TBC
I feel so overwhelmed with grief that I cannot deal with all the changes in my new country.			**Figure:** 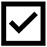 TBC
There are so many things to worry about in my new country that I never have time to grieve.			**Figure:** 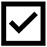 TBC
When I talk about the loss no one understands me.			**Figure:** 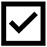 TBC
I am grieving for multiple loved ones at the same time.			**Figure:** 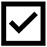 TBC

*Note.* The first column represents the items of the IPGDS cultural supplement. The subsequent columns indicate from which cultural group the item was developed and validated. The items with TBC indicate that these need to be validated in a large sample.

The cultural supplement emerged from a bottom-up qualitative approach. Items were developed from key informant interviews with German-speaking and Chinese-speaking health care workers (Killikelly et al., 2020; Stelzer, Höltge, et al., 2020; Stelzer, Zhou, Merzhvynska, et al., 2020), bereaved migrants and refugees (Killikelly et al., 2021) and Japanese health care professionals (Killikelly et al., 2022). Currently there are several versions of the cultural supplement that are being validated in different cultural groups. The Chinese version has been psychometrically validated in a sample of *n* = 325 Chinese bereaved (Killikelly et al., 2020), the migrant cultural supplement (for bereaved migrant individuals living in a host country) has recently been validated in 121 bereaved migrants (in preparation). A Japanese version and a version for Arabic speaking refugees experiencing ambiguous loss are currently under development.

## Summary of Recent Findings From the Implementation of IPGDS Cultural Supplement

Below we outlined how the cultural supplement of the IPGDS has been used to explore culturally relevant symptoms in different cultural groups. To date the cultural supplement has been used in two main ways 1) to compare and contrast a wide range of possible PGD symptoms between different cultural groups 2) to identify new culturally relevant symptoms of PGD within a cultural group or context. The earliest results from the implementation of the cultural supplement in the Chinese bereaved sample show the value of the supplement. Firstly, an item specific analysis revealed that certain items were endorsed more strongly in the Chinese sample when compared to the German speaking sample. For example, the most strongly endorsed item in the Chinese sample was Item 15 (I constantly look back upon the past relationship) whereas for the German speaking sample it was Item 17 (I feel he/she is beside me). Additionally overall scores on the cultural supplement were higher in the Chinese sample than the German speaking sample, possibly indicating the items were more culturally relevant for the Chinese sample, as expected (Killikelly et al., 2020). Our recent study explored PGD in German-speaking and Chinese samples using a network analysis. We confirmed the presence of a core network of PGD symptoms consisting of yearning and emotional distress in both Swiss and Chinese participants (Stelzer, Höltge, et al., 2020). However, when culturally relevant items were included in the network this improved the predictability of the network for the Chinese sample only, possibly indicating that the cultural supplement yielded a better fit. Important network differences also revealed a strong connection between Item 11 (wish to die to be with the deceased) and Item 14 (searching for the deceased) for Chinese participants that was not found for German-speaking participants. We concluded that separation distress is a particularly relevant therapeutic target for Chinese participants.

In our latest study, 121 migrants to Switzerland completed the standard scale of the IPGDS and the culturally adapted ‘Migrant version’ of the cultural supplement. This version of the cultural supplement was developed from focus groups and interviews with Syrian migrants. New items were developed based on these interviews (e.g., Item 4: The loss shattered my beliefs (i.e., my understanding on how the world should work, spiritual beliefs, religious beliefs), Item 13: I feel like I have completely lost control over my life or over myself and item: 19 I feel disconnected from the new society I live in (e.g. the Country I moved to). Each of these items must be answered in response to the loss of a loved one. To reduce the item list of the cultural supplement, a preliminary analysis of the response rates to each item revealed that the most endorsed items included Item 4 and Item 19. This potentially indicates that the inclusion of these culturally relevant items improved the sensitivity of the Migrant version of the cultural supplement.

## Recommended Methods for Adaptation of the Cultural Supplement

**Figure 1:**
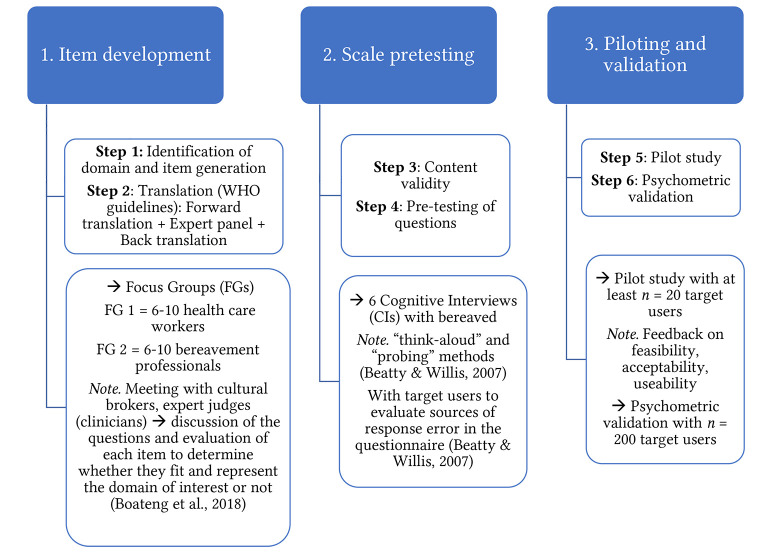
Step by Step Method for Cultural Adaptation of the IPGDS Cultural Supplement

Researchers and clinicians might be interested in developing a cultural supplement for the IPGDS based on their own community and context. In line with this we propose the following steps for adaptation (see Figure 1).

However, first, in cross cultural research there are two important methodological caveats that should be transparently and forthrightly presented, particularly when comparing symptoms of mental disorder between different groups. First, the researchers definition of cultural group should be clearly stated and defined. Cultural group can be defined in different terms with a focus on different features (Ryder et al., 2011). In our research we define cultural group specifically in terms of the features that may intersect with mental disorder and PGD: a group of people who share a common language, regional history, beliefs, patterns of behaviour and values (National Center for Cultural Competence, 2001). This should be measured transparently and systematically through a simple questionnaire. Brief questions of cultural group and identity could be asked for example: what is your country of origin? Which culture influences you the most? How connected do you feel to Western culture? (Killikelly et al., 2020). Second, the effect of unmatched groups should be clearly presented to highlight any possible confounding factors. When possible, cultural groups should be matched in terms of characteristics that might affect the severity of PGD symptoms. For example, loss related characteristics should be similar across groups (e.g., relationship to the deceased, type of loss (natural or unnatural), time since loss). Demographic characteristics such as age, gender, and co-morbid mental health disorders should also be clearly documented.

The first step in the development of a new cultural supplement questionnaire is *item development*. A wide range of possible symptom items are gathered from cultural brokers or key experts such as clinicians or researchers that belong to the cultural group of interest and have key clinical knowledge. For example, ‘free listing’ is a technique used to elicit a large number of possible symptoms (Kumar, 1989; World Health Organisation, 2012). Focus groups may then reduce the number of items to the most highly endorsed and relevant. These items are then translated into the language of the group under study following the WHO’s recommended translation process. The second step is scale pretesting (Boateng et al., 2018). The content validity of newly suggested symptoms can then be established via cognitive interviews with a representative sample of bereaved individuals (e.g., gender, age, type of loss, duration of loss all represented). For information on how to conduct cognitive interviews including the think aloud and probing technique see (Abi Ramia et al., 2018; Drennan, 2003; Prince, 2008). The aim of this step is to ensure that the format and nature of the questions are clear, concise and valid. Finally, the new questions should be piloted in a small sample of intended users. This can be followed by a larger scale psychometric validation study whereby standard psychometric properties of the scale (validity and reliability) are established (see Killikelly et al., 2020).

It should be noted that the cultural supplement should be used alongside the ‘standard scale’ i.e., the 14 items of the ICD-11 PGD definition. This is particularly important when establishing cross cultural prevalence rates, differences and similarities of symptom structure or establishing methodologically robust comparisons across different groups and contexts. The ‘standard scale’ provides the ICD-11 PGD symptom list and could be used in clinical samples for diagnosis. This assumes the universalist approach to mental disorders. The cultural supplement can add to this list for purposes of exploring alternative PGD symptoms and supporting treatment planning. For example, somatic symptoms are not included in the ICD-11 PGD definition, however several different cultural groups have strongly endorsed physical symptoms following bereavement. After assessing with the IPGDS, a clinician may then offer interventions and techniques to alleviate somatic symptoms if these are most distressing symptoms indicated. These somatic symptoms may not be discovered without the wide range of questions covered by the cultural supplement.

## Implications and Future Research

Since 2022 the new ICD-11 is used worldwide in clinics and research settings. Researchers are presented with a unique opportunity to document the impact of the inclusion of a new mental health disorder, PGD on patient experience and clinical outcomes. Additionally, the inclusion of the cultural caveat presents several challenges and opportunities. We invite clinicians and researchers to consider using the IPGDS and the cultural supplement in their clinical and research settings to add to the growing database of literature exploring prolonged grief disorder in different cultural contexts. There are many questions that remain unanswered about the relationship and importance of culture and mental disorder. One outstanding research question concerns the etiology of prolonged grief disorder and its’ location on the universalist versus relativist spectrum. The development and testing of additional cultural supplements in different cultural groups worldwide could help identify which prolonged grief disorder symptoms are universal and which are culturally relative. One hypothesis could be that core symptoms of prolonged grief disorder are universal for example yearning and preoccupation with the deceased, while supplementary symptoms and examples of emotional distress may vary depending on cultural group. Although currently we recommend assigning a PGD diagnosis following the guidance of the IPGDS standard scale and the ICD-11 PGD definition, treatment planning may be enhanced with a person-specific approach. Therefore, the cultural supplement may be a valuable tool to improve clinician-patient relationship and treatment decision making. It is important to note that the cultural supplement is subject to some key limitations. For example, the definition of culture will be specific to the research group, the research question and the sampling method. Researchers and clinicians should provide thorough and transparent information on how they selected participants and the sampling method used to determine the cultural group or context. Additionally, it will be important to clearly document loss related variables, such as the type of loss, time since loss and the nature of the loss (sudden, violent etc.) as this may have a significant impact on the nature and severity of PGD symptoms particularly in different cultural contexts (Djelantik et al., 2020). In conclusion, the inclusion of PGD as a mental health disorder opens the door for further robust, systematic research on the relationship between grief and culture.
